# *Drosophila* Ovipositor Extension in Mating Behavior and Egg Deposition Involves Distinct Sets of Brain Interneurons

**DOI:** 10.1371/journal.pone.0126445

**Published:** 2015-05-08

**Authors:** Ken-ichi Kimura, Chiaki Sato, Masayuki Koganezawa, Daisuke Yamamoto

**Affiliations:** 1 Laboratory of Biology, Hokkaido University of Education, Sapporo Campus, Sapporo, Japan; 2 Division of Neurogenetics, Tohoku University, Graduate School of Life Sciences, Sendai, Japan; Alexander Fleming Biomedical Sciences Research Center, GREECE

## Abstract

Oviposition is a female-specific behavior that directly affects fecundity, and therefore fitness. If a fertilized female encounters another male that she has evaluated to be of better quality than her previous mate, it would be beneficial for her to remate with this male rather than depositing her eggs. Females who decided not to remate exhibited rejection behavior toward a courting male and engaged in oviposition. Although recent studies of *Drosophila melanogaster* identified sensory neurons and putative second-order ascending interneurons that mediate uterine afferents affecting female reproductive behavior, little is known about the brain circuitry that selectively activates rejection versus oviposition behaviors. We identified the sexually dimorphic pC2l and female-specific pMN2 neurons, two distinct classes of *doublesex (dsx)*-expressing neurons that can initiate ovipositor extension associated with rejection and oviposition behavior, respectively. pC2l interneurons, which induce ovipositor extrusion for rejection in females, have homologues that control courtship behavior in males. Activation of these two classes of neurons appears to be mutually exclusive and each governs hierarchical control of the motor program in the VNC either for rejection or oviposition, contributing centrally to the switching on or off of the alternative motor programs.

## Introduction

Mating and oviposition are two major activities that affect the fitness of adult female insects. Mating is a prerequisite for laying fertilized eggs, yet inappropriate mating by fertilized females may reduce their fecundity. The inseminated female who has made decision not to mate again will display post-mating behaviors, i.e., rejection behavior toward the second male and depositing of eggs upon finding an appropriate oviposition site. The female post-mating behaviors are believed to rely on a hard-wired neural system that is postulated to integrate a variety of neural inputs which encode both internal and external information and which activate the neural center for either rejection or oviposition [1]. However, any higher-order neurons that may be involved in this circuitry and their connectivity remain largely elusive, because studying neural circuits at the single cell level is technically demanding.

For example, in the cabbage white butterfly *Pieris rapae*, mated females raise their abdomen to reject courting males, whereas virgin females accept and mate with the males [2]. The behavioral change between the virgin-type and mated-type of these female butterflies is triggered during copulation by stretch receptor afferents that originate in the bursa copulatrix. Incoming sperm will cause this structure to expand and generate in sensory neurons ascending impulses toward the thoracic ganglia [2]. Although serotoninergic involvement has been suggested [3], the central neurons contributing to this post-mating behavior have not been identified. In orthopteran insects, however, interneurons contributing to the central pattern generator (CPG) for oviposition have been identified in the terminal abdominal ganglion [4]. This CPG for oviposition is activated by disinhibition mediated by descending projections from interneurons located in the brain; transection of the cervical connectives releases the oviposition program. However, the identity of these inhibitory interneurons remains unknown [5].

The fruit fly *Drosophila melanogaster* provides an unparalleled model for studying neural circuitry that mediates innate behaviors such as mating and oviposition at the resolution of a single cell. Genetically engineered tools allow selective activation or inactivation and labeling of individual neurons [6 for review] so that a causal link between single neurons and a given behavior can be evaluated through controlled experiments. By taking advantage of these neurogenetic tools in *Drosophila*, we aimed to elucidate the higher-order neurons involved in female mate refusal and oviposition.

The courtship behavior of receptive virgin *D*. *melanogaster* females is characterized by an initial decamping from a courting male, which is followed by slowdown of locomotion upon receiving sustained courtship from a male, and finally acceptance of the male [7]. Recently mated females reject courting males through a variety of actions such as decamping, flicking their wings, kicking the approaching male, or extrusion of the ovipositor [8]. Thus, copulation switches female behavior from acceptance to rejection. Another remarkable change in female behavior after copulation is a dramatic acceleration of egg laying. A mated female that has found a suitable site for oviposition will bend her abdomen downward until it is nearly perpendicular to the substrate and insert the ovipositor into the substrate before performing a series of back-and-forth movements to expel a single egg that is inserted into the substrate [9]. This behavior is called the ovipositor motor program [9]. After depositing an egg, the female grooms its ovipositor with its hindlegs and remains immobile for a while [9].

We were interested in the common use of the ovipositor in different contexts, especially mate refusal and oviposition; this prompted us to examine how the nervous system selects the two motor programs involved in ovipositor movement.

In this study, we focus on *dsx*-expressing neurons, as they are known to play major roles in female-specific reproductive functions [10, 11, 12, 13, 14], although some neurons that do not express *dsx* are also critically involved in female reproductive behavior [12, 15, 16]. We identify two groups of brain neurons that govern hierarchical control of the mate refusal or oviposition motor program in the VNC, contributing centrally to the choice of alternative motor programs, i.e., the program for mate refusal and that for oviposition behavior. The present work unravels the sophisticated neural network underlying the female choice of reproductive strategy and paves the way for the study of the physiological mechanisms involved in other simple decision-making processes.

## Materials and Methods

### Identification of neurons

*dsx* ^*GAL4*^ *(G)* (a gift from S. Goodwin at the University of Oxford in England) was used to drive *UAS-mCD8*::*GFP* in labeling *dsx-GAL4*-expressing neurons. The brain was dissected, fixed with 3.7% formaldehyde (30 min), washed in PBS-Tx, and reacted with an anti-GFP antibody (1:500; Molecular Probes) or anti-mCD8 antibody (1:500; Caltag, Burlingame, VT) and the monoclonal antibody nc82 (1:200; a gift from A. Hofbauer). Staining was visualized by Cy2 and Cy3 (1:500, Jackson Immuno-Research, West Grove, PA). To determine the projection patterns of *dsx-GAL4*-expressing neurons, we employed the lineage-based Mosaic Analysis with a Repressible Cell Marker (MARCM), which allows labeling and manipulation of a small set of cells that are clonally related [17]. MARCM was used in flies with the following genotypes: *y hs-flp*; *G13 UAS-mCD8*::*GFP/G13 Tub-GAL80*; *dsx* ^*GAL4*^ *(G)/+* or *y hs-flp/+ (Y)*; *G13 UAS-mCD8*::*GFP/G13 Tub-GAL80*; *dsx* ^*GAL4*^ *(G) /UAS-dTrpA1*. Here *G13* represents a Flippase (Flp) recognition target (FRT) site, on which Flp acts to induce recombination events. Chromosomal recombination was induced to generate MARCM clones by applying heatshock(s) at 37°C for 1 h (once or 4 times), beginning 24 h after egg-laying. Treated animals were subjected to brain histology within 10 days of eclosion, as described above. Approximately 2000 individuals were examined for MARCM labeling of *dsx-GAL4*-expressing neurons. To observe neurons in the *dsx* mutant background, *dsx* ^*GAL4*^ *(B)* (donated by Bruce Baker, Janelia Farm, Ashburn, VA) was used to drive a marker transgene. Note that *dsx* ^*GAL4*^ *(G)* produces functional Dsx proteins [18] whereas *dsx* ^*GAL4*^ *(B)* does not [19].

### Behavioral assays after activating *dsx* neurons *en masse*, within or outside the brain

**Fig 1:**
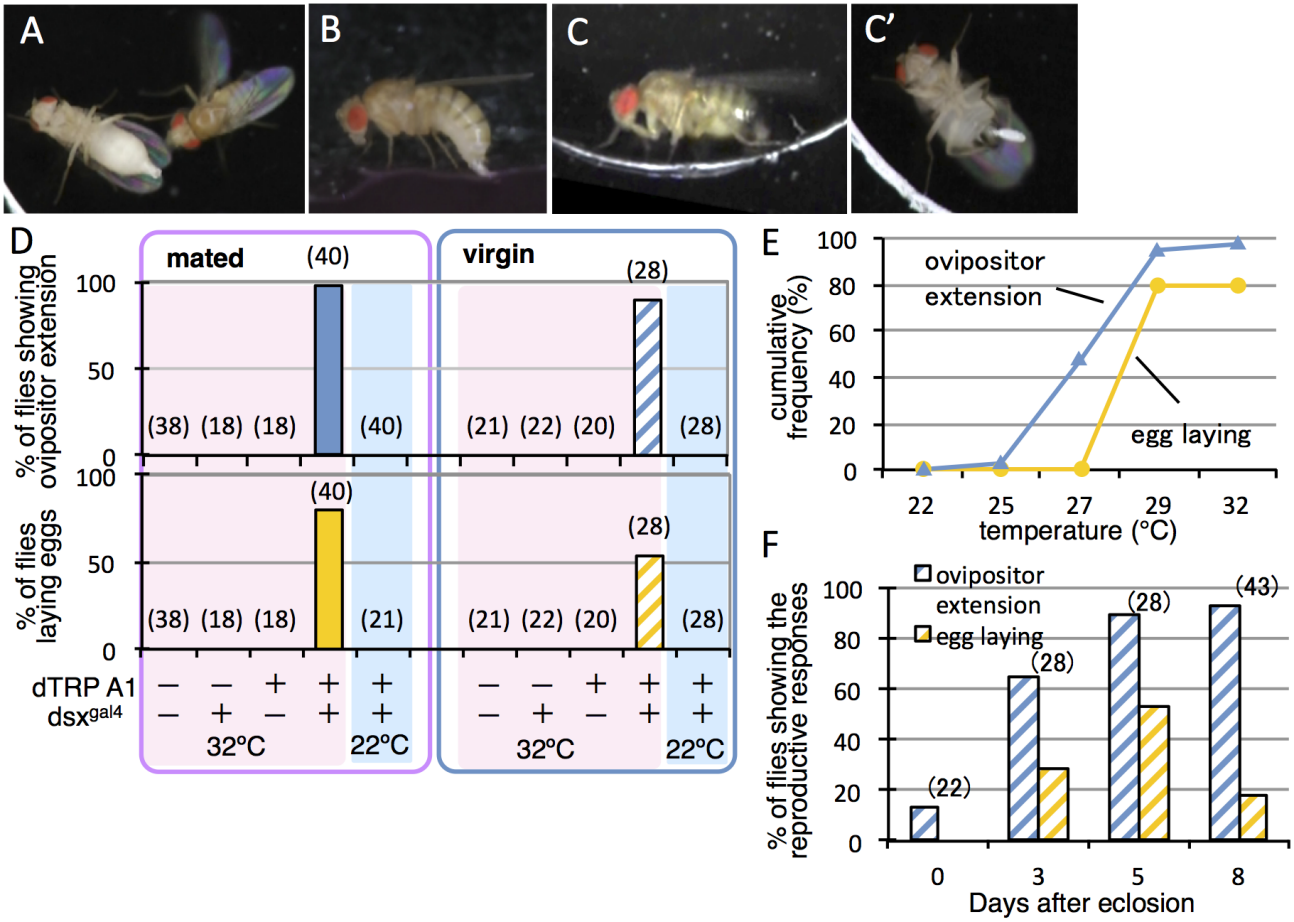
Activation of *dsx-GAL4*-expressing neurons in females induces oviposition-type extension of the ovipositor and egg-laying. (**A**) Extrusion of the ovipositor in response to male courtship in mated wild-type females. (**B**) Egg-laying in mated wild-type females. (**C, C’**) The oviposition posture with ovipositor extension (**C**) and egg-laying (**C’**) was artificially induced in mated females that express dTrpA1 under the control of *dsx* ^*GAL4*^ *(G)* by a temperature increase to 29°C. (**D**) The proportion of mated (left-hand graph) and virgin (right-hand graph) flies that engaged in the ovipositor extension or egg-laying upon temperature increases up to 32°C was compared among the 4 genotypes indicated at the bottom. (+) and (–) indicate the presence or absence of *dsx* ^*GAL4*^ *(G)* and *dTrpA1* in the fly groups examined. (**E**) Cumulative plots of the number of *dsx* ^*GAL4*^ *(G)>dTrpA1* mated females (n = 40) that exhibited ovipositor extension or egg-laying when the ambient temperature was increased from 22°C to 32°C. (**F**) The proportion of virgin flies (*dsx* ^*GAL4*^ *(G)>dTrpA1*) exhibiting ovipositor extension or egg-laying upon a temperature increase to 32°C was compared at different ages: 0 (within 24 h), 3, 5, and 8 days after eclosion. The numbers shown in parentheses (D, F) indicate the number of flies examined.

Flies of the genotype *G13 UAS-mCD8*::*GFP*; *dsx* ^*GAL4*^ *(G) UAS-dTrpA1 /TM6b Hu Tb (dsx* ^*GAL4*^ *(G)>dTrpA1)* for activating *dsx* neurons *en masse*, flies of the genotype *UAS>stop>dTrpA1-myc/Otd-nsl*:*FLP; dsx* ^*GAL4*^ *(G) /+* for activating *dsx* neurons in the brain, and flies of the genotype of *Tub>stop>GAL80/Otd-nsl*:*FLP; dsx* ^*GAL4*^ *(G) UAS- dTrpA1/ +* for activating *dsx* neurons outside the brain were raised at 22°C under constant light conditions. Each female was transferred to a glass vial (8 mm in diameter, 20 mm in height) containing fresh food, and they were maintained at 22°C until required for use. Behavioral assays were performed for females 5 days after eclosion, except for the experiment shown in Fig 1F. For the behavioral assays, each female fly was isolated in a vial and placed on a cooling plate. The temperature was increased in 2-min intervals from 22°C to 25°C, 27°C, 29°C, and 32°C; the presence or absence of any behavior at each temperature step was recorded. For experiments with mated females, the females were placed in a vial with two male flies (about 5 days after eclosion) for a few days, and the females were considered to have mated when offspring larvae were observed in the vial. Decapitation was performed for *dsx* ^*GAL4*^ *(G)>dTrpA1* flies on the fourth day after eclosion, and the behavioral response to temperature increases was examined 1 day after decapitation.

### Behavioral MARCM

The genotypes of the flies used were *y hs-flp/+(Y)*; *G13 UAS-mCD8*::*GFP/G13 Tub-GAL80*; *dsx* ^*GAL4*^ *(G)/UAS-dTrpA1*. To generate MARCM clones, chromosomal recombination was induced by applying 4 heatshocks at 37°C for 1 h, with 5-h intervals, beginning 24 h after egg-laying. The treated animals were maintained at 22°C until required for use. The females emerged 5–8 days after eclosion and were paired with wild-type (CS) males and allowed to mate freely. The test females were transferred individually to an arena (10 mm in diameter, 2 mm in height) and observed for the presence or absence of ovipositor extension or oviposition response for 2 min at 35°C. This trial was repeated 3 times at 2–3-h intervals, and a female was judged positive for a behavior when she exhibited it at least once during the entire session. The temperature used to activate neurons in some behavioral MARCM experiments was 35°C because of the need to induce behavioral responses quickly. We ascertained that the behaviors of wild-type females without any transgenes were normal at this temperature during the observation period of 2–3 min, except for the elevation of locomotor activity. When the female exhibited the oviposition-type extension of the ovipositor and/or laid an egg at least once in 3 trials, she was scored as a “Responder-O” fly. When the female exhibited the mating-type extrusion of the ovipositor, she was scored as a “Responder-M” fly. The females that showed no reproductive response were classified as Responder-N flies (“N” stands for neither mating nor oviposition). The brains were dissected from responder and non-responder females and subjected to histological examination to determine which cells were mCD8::GFP-positive, as described above.

The male mosaic flies were subjected to single male assays 5–8 days after eclosion. Male flies were placed individually in an arena (10 mm in diameter and 2 mm in height) and were observed for 2 min at 35°C in order to identify and collect flies that exhibited wing extension and vibration. After resting for at least a few hours, flies that were positive for wing displays were subjected to video recordings of behavior for 3 min at 35°C to determine whether they exhibited any additional courtship action, i.e., tapping, licking, or abdominal bending for copulation. When a male fly showed any of these courtship actions, it was classified as a “Responder-C” fly. The flies showing only wing display were included in the “non-Responder” category, together with flies that did not show even a wing display. We reasoned that exclusion of flies exhibiting only wing display will enrich the flies in which activation of brain neurons is responsible for induced courtship, because wing displays can be initiated by activation of thoracic neurons alone with no involvement of brain neurons [20]. The brains were dissected from flies classified as “Responder-C” and “non-Responder” flies for immunostaining with the anti-GFP or anti-mCD8 antibody in order to identify dTrpA1-expressing cells and with the monoclonal nc82 antibody for neuropil staining. Stacks of optical sections of 1 or 2 μm were obtained with a Leica TCS SPE confocal microscope using LAS-AF software and were processed with Adobe Photoshop.

## Results

### Female reproductive behaviors are induced by activation of all *dsx* neurons

Under natural conditions, the recently mated female occasionally extends the ovipositor straight along her body axis (anterior-posterior axis) toward the courting male (Fig 1A, S1 Movie), who usually positions himself behind the female [21]. During the ovipositor extension, the female abdomen was width-compressed throughout its entire length. We refer to the ovipositor extension as mating-type extrusion when it was protruded horizontally along the female body axis. A mated female that has found a suitable site for oviposition will lower and bend her abdomen downward and insert the ovipositor into the substrate before performing movements to expel an egg that is inserted into the substrate (Fig 1B, S2 Movie) [9]. Unlike the extrusion for rejection, the female narrowed the abdomen only at its posterior part.

We first examined the effects on female behaviors of forced activation of *dsx*-*GAL4*-positive neurons *en masse* with dTrpA1, a warmth-sensitive channel. In both virgin and mated females, upon artificial activation of all *dsx-GAL4* neurons via dTrpA1, the ovipositor was extended backward first, usually followed by downward bending of the abdomen, and even by egg ejection in some cases (Fig 1C and 1C’, S3 Movie, S4 Movie); egg ejection was induced in approximately 80% of mated females tested and even in 50% of virgin females (Fig 1D). Without activation via dTrpA1, no extrusion was observed even in mated females, when they were placed alone in the chamber without presenting any target for rejection display, such as a courting male. In the presence of a courting male, by contrast, dTrpA1-mediated neural stimulation at 29°C induced the oviposition posture with ovipositor extension in the mated female, who, in addition voluntarily extruded their ovipositors toward the male (S5 Movie). Under our experimental conditions, no egg ejection was observed even in mated females unless they were stimulated via dTrpA1, presumably because no medium suitable for oviposition was provided. Egg ejection was typically detected when the females lowered and/or bent the abdomen; interestingly, egg ejection often occurred even before they bent the abdomen. Note that egg ejection in response to male courtship has been observed, albeit rarely, in unreceptive females under natural conditions [8]. Thus the mechanism for egg ejection *per se* is likely separable from the mechanism for adopting an oviposition posture or that for exhibiting extrusion, although egg laying is normally concomitant with the oviposition posture. We consider that the extension of the ovipositor accompanied by ventral bending of the abdomen is an element of oviposition behavior (we define this female action as the “oviposition posture”), which may or may not result in egg deposition. We cannot exclude the possibility, however, that extrusion initiated as an element of the rejection behavior program turns to ovipositor extension in the oviposition behavior program, ultimately resulting in egg deposition.

In the present experiments, ovipositor extension associated with the oviposition posture, i.e., oviposition-type extension, was induced at temperatures lower than those required for egg deposition (Fig 1E). We found that, on the day of eclosion, virgin female flies barely adopt the oviposition-type extension upon dTrpA1-mediated activation of *dsx*-*GAL4*-expressing cells, whereas a significant proportion of manipulated females exhibited these behaviors with egg deposition 3 days after eclosion (Fig 1F), presumably reflecting their sexual maturation [22, 23]. The ovipositor-related motor pattern generators are known to be located in the abdominal segments of the VNC [9]. To determine whether the VNC alone can generate mating-type extrusion and oviposition-type extension, *dsx-GAL4*-positive cells in the VNC were activated via dTrpA1 in decapitated virgin females with an intact VNC. We found that nearly half of the decapitated females showed the oviposition-type extension (nine out of 17 flies tested) and even egg ejection (seven out of 17 flies). We conclude that *dsx-GAL4*-expressing neurons in the VNC alone can produce the motor patterns necessary for oviposition.

### Are brain neurons involved in extrusion and oviposition?

**Fig 2:**
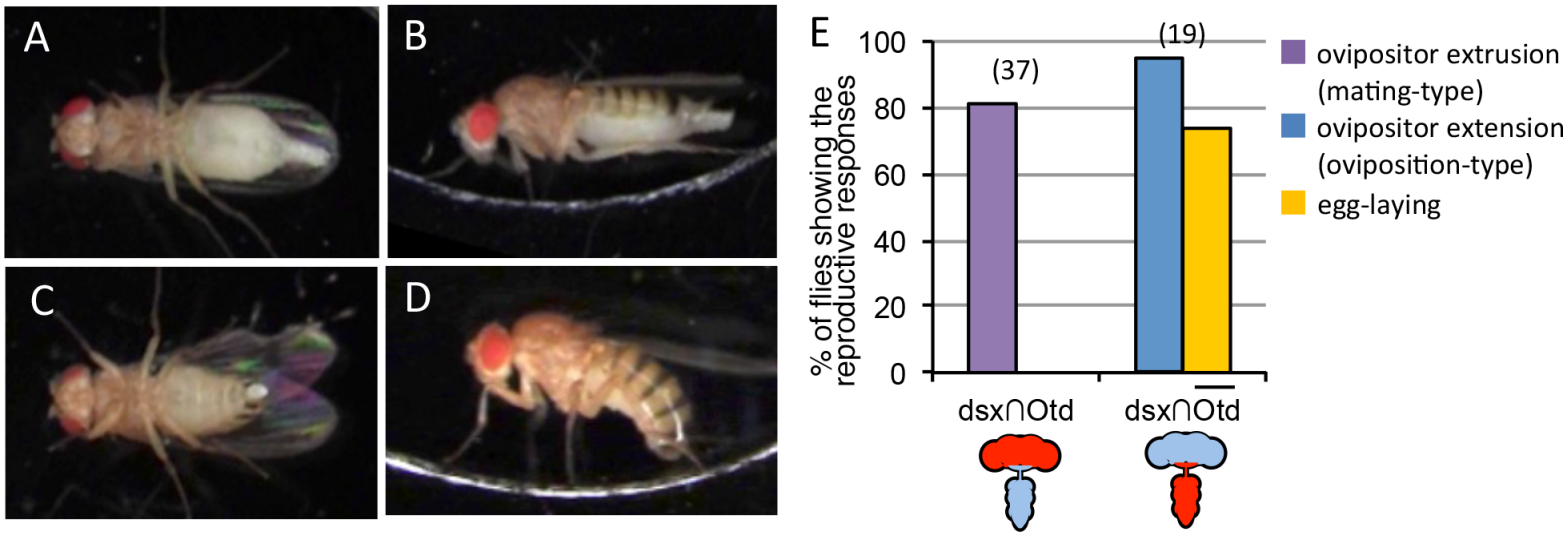
Activation of *dsx-GAL4* neurons in the brain induced mating-type extrusion and that outside the brain induced oviposition-type extension/egg-laying. (**A, B**) Brain-restricted activation of *dsx-GAL4* neurons induced mating-type extrusion of the ovipositor in a female with the genotype of *UAS>stop>dTrpA1-myc/Otd-nsl*:*FLP; dsx* ^*GAL4*^ *(G) / +* by an increase in temperature to 32°C. The ventral view (**A**) and lateral view (**B**) of a mated female displaying mating-type extrusion are shown. (**C, D**) Activation of *dsx-GAL4* neurons outside the brain induced oviposition-type extension/egg ejection in a female with the genotype of *Tub>stop>GAL80/Otd-nsl*:*FLP; dsx* ^*GAL4*^ *(G) UAS- dTrpA1 / +* by an increase in temperature to 32°C. The ventral view (**C**) and lateral view (**D**) of a mated female displaying oviposition-type extension are shown. (**E**) The proportion of females that exhibited mating-type extrusion/egg-laying (left) or oviposition-type extension/egg-laying (right) upon a temperature increase to 32°C. The numbers shown in parentheses (**E**) indicate the number of flies examined. The drawings below the graph are nervous systems and the red colored region represents the activating sites by dTRPA1.

Although the decapitated females were able to show oviposition-type extension and egg ejection upon activation of *dsx-GAL4* neurons, this does not necessarily mean that the brain is indispensable for normal regulation of these behaviors. To clarify the possible involvement of brain neurons in the control of mating-type extrusion and oviposition-type extension, we examined the effect on female behavior of stimulating *dsx-GAL4* neurons only within the brain (Fig 2A, 2B and 2E) or only outside the brain (Fig 2C, 2D and 2E), by employing the brain-specific *Otd-FLP* [24] to activate otherwise inert *UAS>stop>dTrpA1* or to repress GAL4 via otherwise inert *Tub>stop>GAL80*.

Remarkably, brain-restricted activation of *dsx-GAL4* neurons induced mating-type extrusion, but not the oviposition posture and egg ejection, in mated females (Fig 2E, S6 Movie). This result demonstrates that the brain at least plays a role in the initiation of mating-type extrusion. In contrast, activation of *dsx-GAL4* neurons outside the brain successfully induced the oviposition posture in mated females (Fig 2C, 2D and 2E, S7 Movie) but rarely induced the mating-type extrusion. Notably, adoption of the oviposition posture was here accompanied by egg ejection in mated females (Fig 2E). These results support the notion that *dsx-GAL4*-expressing neurons in the VNC alone can produce the motor patterns necessary for oviposition. It remains to be determined whether reciprocal inhibitory pathways between the brain and abdominal ganglia function to ensure the all-or-none induction of ovipositor extrusion for rejection and oviposition behavior.

### Anatomical identification of *dsx-GAL4*-expressing brain neurons

**Fig 3:**
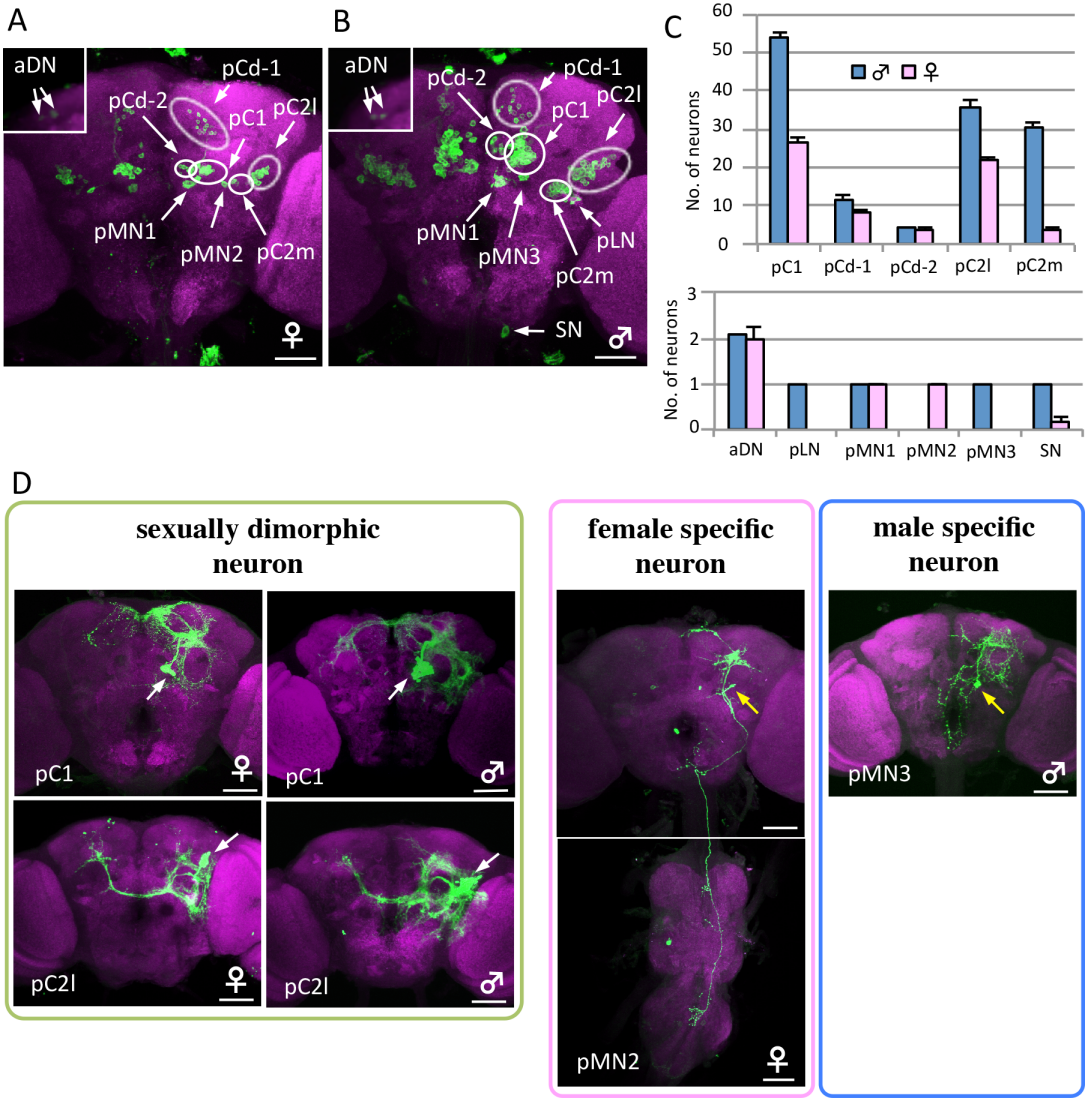
Sex differences in *dsx-GAL4*-expressing neurons. (**A**, **B**) Posterior view of a female (**A**) and male (**B**) brain in the flies expressing *UAS-mCD8*::*GFP* under the control of *dsx* ^*GAL4*^ *(G)*. Islets in **A** and **B** are shown in anterior view. The genotype of flies used is *y hs-flp;G13 UAS-mCD8*::*GFP;dsx* ^*GAL4*^ *(G)*. (**C**) The number of neurons contained in 11 *dsx-GAL4*-expressing neuron clusters was compared between the female and male brain. Values represent the mean ± s.e. (n = 12). (**D**) Examples of sex differences in *dsx-GAL4*-expressing neuron clusters. The somata of neuron clusters and single neurons indicated as MARCM clones are shown using white and yellow arrows, respectively. The brains were stained with anti-GFP (or anti-mCD8) antibodies (green) and nc82Mab (magenta). The scale bars represent 50 μm.

Although the above results seem to suggest that the brain is involved only in the control of mating-type extrusion with no role in oviposition-type extension of the ovipositor, there are reports that the brain stimulates [25] as well as inhibits [26] oviposition. Because the VNC-brain interplay seems to be mediated by multiple excitatory as well as inhibitory pathways, manipulation of *dsx-GAL4* neurons *en masse* inevitably results in complications due to simultaneous activation of these counteracting mechanisms. To circumvent this problem, we employed the lineage-based MARCM, which allows labeling and manipulation of a small set of cells that are clonally related [17]. As a first step, we used MARCM to identify all *dsx-GAL4*-positive neurons in the brain. In total, 140 and 280 neurons were *dsx-GAL4*-positive in the female and male brains, respectively (Fig 3A–3C and S1 Table; cf. Refs. [18, 19]). The number of *dsx*-positive cells in the male brain is consistent with that reported by Rideout et al. [18], but unexpectedly, that in the female brain is twice as large as in Rideout et al. [18]. In contrast to preceding works [18, 19], we were able to separate neurites of different cell clusters as benefitted by clonal labeling of individual neural clusters. Based on the location of their somata and projection patterns, 11 groups of *dsx-GAL4*-positive neurons were unequivocally identified in the brain (S1 Fig, S1 Table). Included in these 11 groups were the female-specific pMN2 cluster and the 3 male-specific clusters SN (suboesophageal neurons), pLN, and pMN3 (Fig 3D). Neurons in the pC1, pC2l, pMN1, and aDN clusters had sexually dimorphic projection areas (Fig 3D and S1 Fig). There were approximately 300 *dsx-GAL4*-positive neurons in the abdominal ganglia of the VNC, which were densely packed and were usually co-labeled under our MARCM conditions, making it difficult to identify them individually. Therefore, we pooled these neurons as the VNC cell group to analyze the correlation between behavior and activated cell groups.

### Functional identification of higher-order neurons that initiate extrusion and oviposition

**Fig 4:**
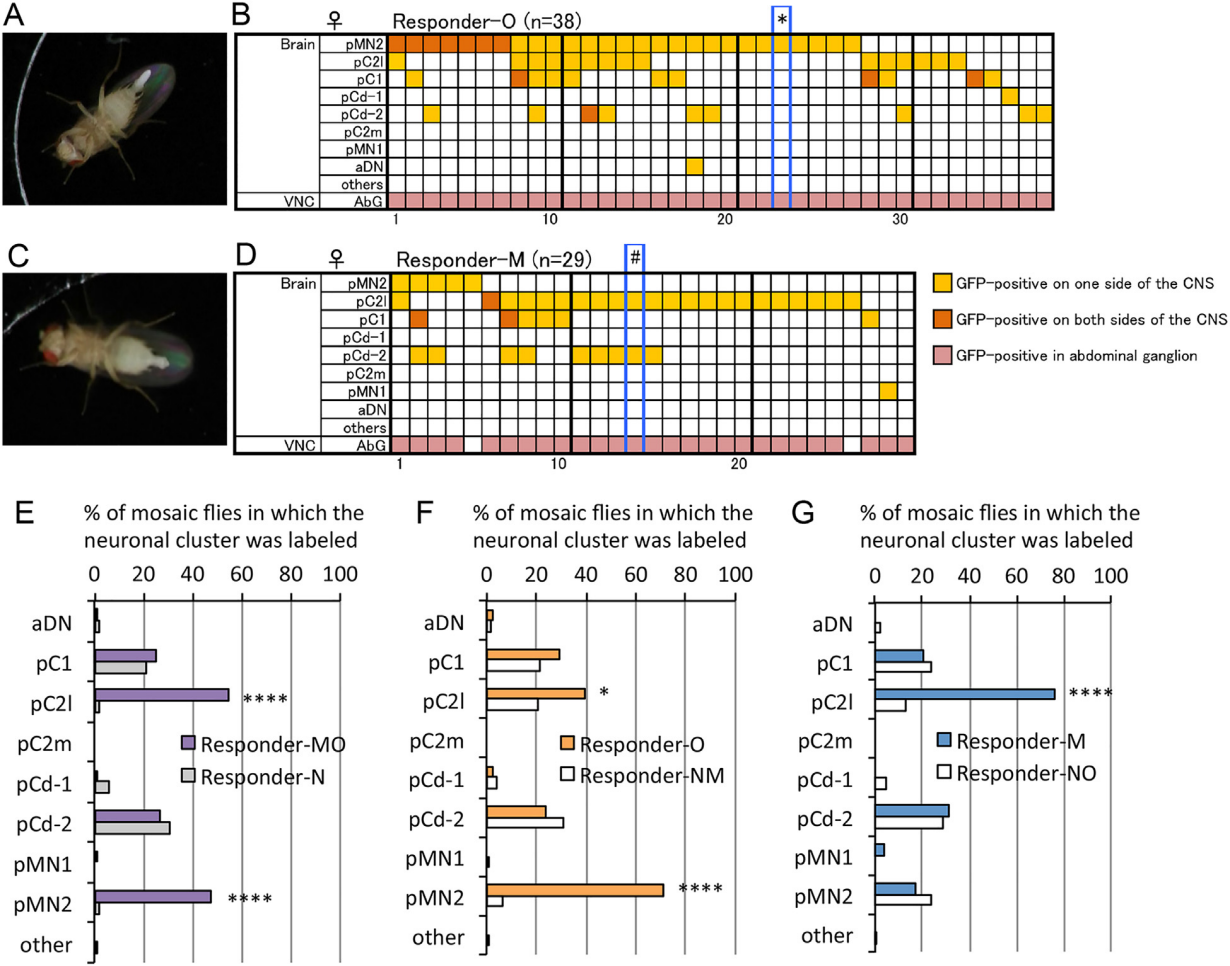
Behavioral MARCM identifies the neurons that initiate mating-type ovipositor extrusion or oviposition-type extension/egg ejection. (**A-D**) Oviposition posture and egg-laying (**A**) and mating-type extrusion (**C**) observed in flies carrying MARCM clones that express dTrpA1 in *dsx-GAL4*-positive cells. (**B, D**) Labeling pattern of GFP expression in MARCM females. Each vertical column represents scores for a single fly. Neuron classes indicated in the left-side column are described in Fig 3A, S1 Fig and S1 Table. * in **B** indicates that the images shown in Fig 4A, S3A and S3B Fig, and S8 Movie are for this fly, and # in **D** indicates that the images shown in Fig 4C, S3C and S3D Fig, and S9 Movie are for this fly. (**E**) The proportion of flies that carried mCD8::GFP-labeled dTrpA1-expressing cells in the indicated cluster is compared between the non-responder group (Responder-N, n = 84) and responder group (Responder-MO, n = 67). The responder group included flies that exhibited either oviposition-type extension/egg-laying or mating-type extrusion. (**F)** The proportion of flies that carried mCD8::GFP-labeled dTrpA1-expressing cells in the indicated cluster is compared between the fly group that responded with oviposition-type extension/egg-laying (Responder-O, n = 38) and the fly group that did not show oviposition-type extension/egg laying (Responder-NM, n = 113). (**G**) The proportion of flies that carried mCD8::GFP-labeled dTrpA1-expressing cells in the indicated cluster is compared between the fly group that responded with the mating-type extrusion (Responder-M, n = 29) and the fly group that did not show mating-type extrusion (Responder-NO, n = 122). * p < 0.05 and **** p < 0.0001, by Fisher’s exact probability test.

We generated dTrpA1-expressing MARCM clone cells in fertilized female flies and observed their behaviors in elevated temperatures to select flies that extended the ovipositor in response to the temperature increase. We observed their behavior in three successive sessions at 2–3-h intervals, and females that did not respond to any of the three temperature increases with mating-type extrusion, adoption of the oviposition posture, or egg ejection were classified as Responder-N females (“N” stands for neither mating nor oviposition). Females that exhibited mating-type extrusion, that adopted the oviposition posture, or that ejected an egg at least once were classified as Responder-MO females (“MO” stands for mating or oviposition). In the subsequent analysis, the Responder-MO flies were further classified into two groups, Responder-O and Responder-M. Females that exhibited the oviposition posture or that expelled an egg at least once in 3 trials were classified as Responder-O females (“O” stands for oviposition; Fig 4A, S8 Movie). Note that a few mosaic females that ejected an egg while projecting the ovipositor horizontally (i.e., mating-type extrusion) were included in the Responder-O group, assuming that, in these cases, execution of the oviposition program was aborted so that abdominal bending failed to accompany the ovipositor extension. Females that extruded the ovipositor horizontally without expelling an egg were classified as Responder-M flies (“M” stands for mating-type extrusion; Fig 4C, S9 Movie). In our search for the neurons responsible for oviposition, we pooled Responder-N and Responder-M (Responder-NM) flies and compared them with Responder-O flies. Conversely, in searching for the neurons responsible for mating-type extrusion, we pooled Responder-N and Responder-O flies (Responder-NO) and compared their labeling pattern with that for Responder-M flies. For all groups, the brain was dissected from females (S2 Table) to identify neurons that were MARCM clones and expressed mCD8::GFP, which is chromosomally linked with *dTrpA1* (Fig 4B and 4D, S2 Fig). If a particular neural cluster can induce mating-type extrusion or oviposition-type extension/egg ejection, then this cluster will be labeled more frequently in the Responder-M or Responder-O group than in the Responder-NO or Responder-NM group.

Flies bearing MARCM clones in either pC2l or pMN2 were highly significantly enriched in the groups exhibiting either mating-type extrusion or oviposition-type extension/egg ejection (Responder-MO), compared with the Responder-N group (Fig 4E). No other *dsx-GAL4*-positive clusters in the brain revealed a correlation between dTrpA1 expression and temperature-induced behaviors (Fig 4E). Intriguingly, dTrpA1 expression was strongly correlated with oviposition-type extension/egg ejection in pMN2 at the statistically significant level of *P*<0.0001, but only weakly so in pC2l at P<0.05 (Fig 4F). In contrast, dTrpA1 expression was correlated with mating-type extrusion (without egg deposition) in pC2l, but not pMN2 (Fig 4G). Therefore, we consider that pC2l and pMN2 were responsible for the induction of mating-type extrusion and oviposition-type extension/egg ejection, respectively. However, a possible contribution of the VNC neurons to the behavioral effects remains to be determined, because, in the current MARCM experiment, all of the Responder-O flies had some labeled cells in the VNC (Fig 4B). The correlation between pMN2 activation and oviposition-type extension/egg ejection and that between pC2l activation and mating-type extrusion (without egg deposition) were readily detected when clones doubly positive for pMN2 and pC2l were excluded from the analysis (S3 Table). We observed that approximately 81.8% of flies that were positive for pMN2 and negative for pC2l adopted the oviposition-type extension/egg ejection without showing the mating-type extrusion, whereas approximately 77.8% of flies that were positive for pC2l and negative for pMN2 displayed mating-type extrusion without showing the oviposition-type extension (S3 Table). Females are unable to deposit an egg if the uterus does not harbor an egg from the preceding ovulation, even when they are exposed to high temperatures that are sufficient for activating the neural circuits controlling such behaviors [27]. This is a likely reason why some mosaic females that were positive for pMN2 did not deposit eggs when they adopted the oviposition posture with the ovipositor extension. We obtained a mosaic female harboring pC2l clones bilaterally without a pMN2 clone (S3E and S3F Fig). In this female, a temperature increase should act on pC2l bilaterally and thus be most effective in driving relevant motor programs; this female showed mating-type extrusions without egg deposition. Taking these observations into account, we conclude that pMN2 exclusively executes oviposition-type extension/egg ejection, whereas pC2l is dedicated to activation of mating-type extrusion.

### Male-specific DsxM eliminates the female-specific pMN2 neuron

**Fig 5:**
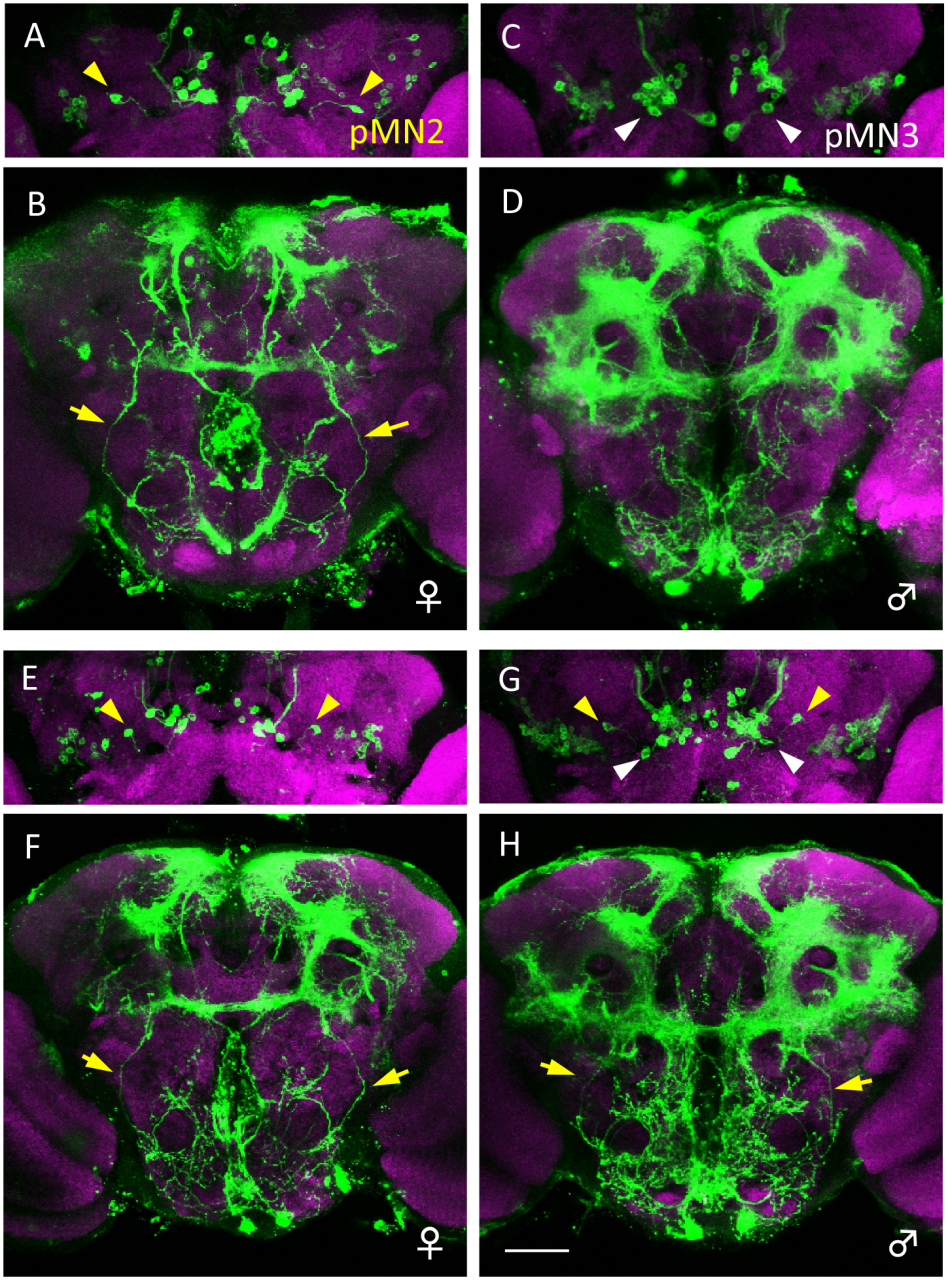
Ectopic formation of female-specific pMN2 in the male brain by artificial expression of the cell death inhibitor p35 in *dsx*-expressing neurons. (**A, B**) A pair of cell bodies of female-specific pMN2 neurons (yellow arrowheads in **A**) and their neurites (yellow arrows in **B**) are present in a wild-type female of *y hs-flp/+*; *UAS-mCD8*::*GFP/+; dsx* ^*GAL4*^ *(G)/+*. (**C, D**) A pair of cell bodies of male-specific pMN3 neurons (white arrowheads in **C**) are present in a wild-type male of *y hs-flp/Y*; *UAS-mCD8*::*GFP/+; dsx* ^*GAL4*^ *(G)/+*. (**E-H**) A pair of cell bodies of pMN2 (yellow arrowheads in **G**) and its neurites (yellow arrows in **H**) are labeled together with pMN3 neurons (white arrowheads in **G**) in the brain of a male fly in which cell-death has been blocked. The fly genotype is *y hs-flp/Y*; *UAS-mCD8*::*GFP/UAS-p35; dsx* ^*GAL4*^ *(G)/+*. In the female of *y hs-flp/+*; *UAS-mCD8*::*GFP/UAS-p35; dsx* ^*GAL4*^ *(G)/+*, the cell bodies of pMN2 neurons and their neurites are observed (yellow arrowheads in **E** and yellow arrows in **F**, respectively). Brains were doubly stained with anti-GFP (green) and nc82 mAb (magenta). The scale bar represents 50 μm.

**Fig 6:**
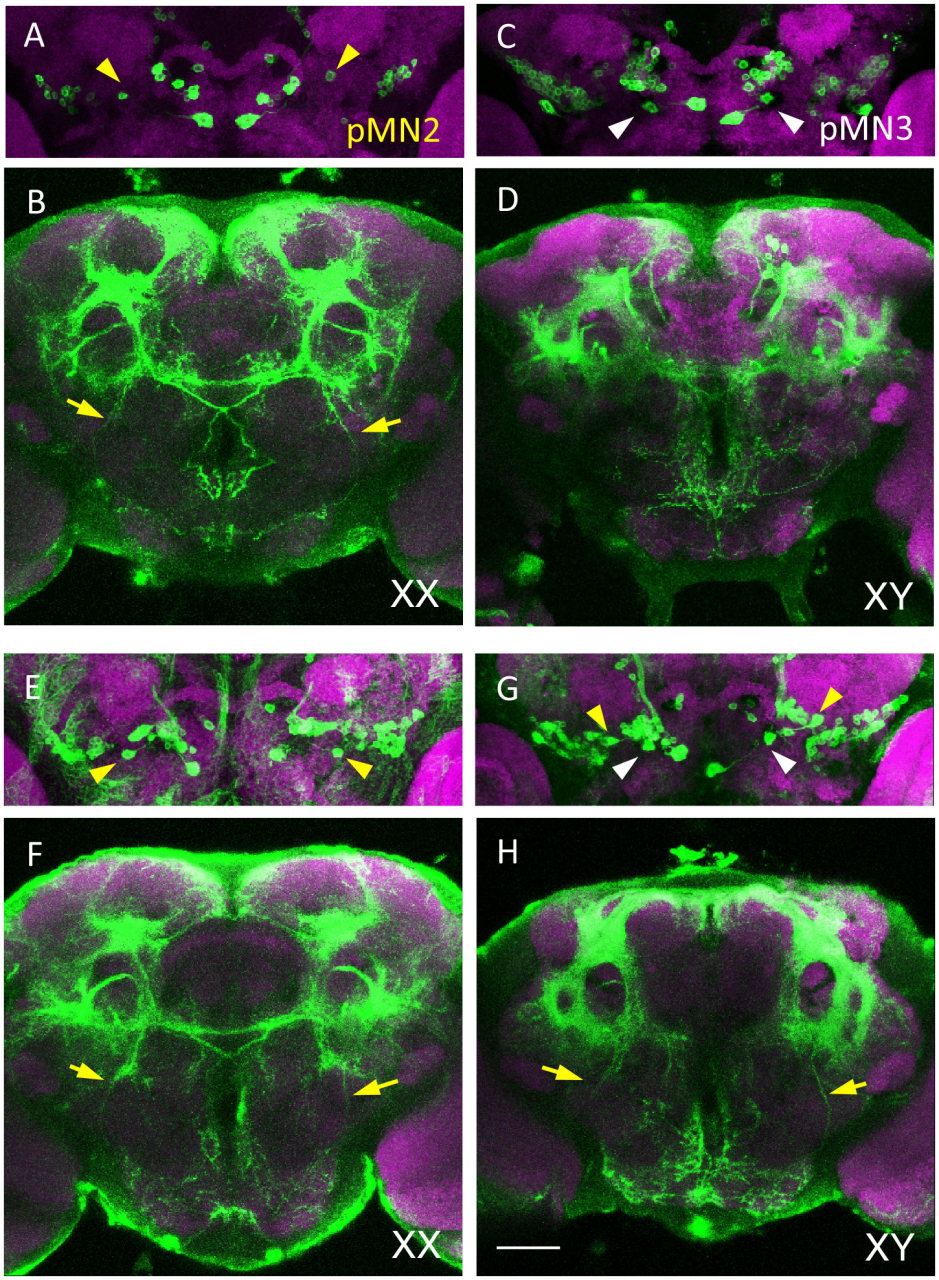
*dsx*-expressing neurons in *dsx* mutants. (**A, B**) A pair of cell bodies of female-specific pMN2 neurons (yellow arrowheads in **A**) and their neurites (yellow arrows in **B**) are present in a control XX female of *UAS-mCD8*::*GFP /+; dsx* ^*GAL4*^ *(B)/TM6b*. (*dsx* ^*GAL4*^ *(B)*; the *GAL4* knock-in null allele of the *dsx* gene was generated by the Baker group at Janelia Farm Research Campus (Ashburn, VA)). (**C, D**) A pair of cell bodies of male-specific pMN3 neurons (white arrowheads in **C**) are present in a wild-type male (XY) of *UAS-mCD8*::*GFP/+; dsx* ^*GAL4*^ *(B)/TM6b*. (**E, F**) In the female (XX) of *UAS-mCD8*::*GFP/+; dsx* ^*GAL4*^ *(B)/dsx* ^*15*^, the cell bodies of pMN2 neurons and their neurites are observed (yellow arrowheads in **E** and yellow arrows in **F**, respectively). (**G, H**) A pair of cell bodies of pMN2 (yellow arrowheads in **G**) and its neurites (yellow arrows in **H**) are seen ectopically in addition to male-specific pMN3 (white arrowheads in **G**) in the brain of a male fly (XY) with the genotype *UAS-mCD8*::*GFP/+; dsx* ^*GAL4*^ *(B)/dsx* ^*15*^. Brains were doubly stained with anti-GFP (green) and nc82 mAb (magenta). The scale bar represents 50 μm.

It is known that cell death eliminates the male-specific *dsx*-expressing P1 cluster from the female brain by the action of the female-specific form of the Dsx protein, DsxF [28]. This raises the question of whether the female-specific *dsx*-*GAL4*-positive cells are eliminated from the male brain by cell death. We observed that this is indeed the case; female-specific pMN2 was ectopically produced in the male brain when the cell death inhibitor p35 was artificially expressed under the control of *dsx* ^*GAL4*^ *(G)* (Fig 5). Furthermore, the male brains contained both pMN2 and pMN3 (Fig 5), which indicated that the female-specific pMN2 and the male-specific pMN3 are not homologous neurons. To determine whether male-specific cell death of pMN2 involved Dsx, we examined whether pMN2 was present in the brains of *dsx*-null females and males. We found that female-specific pMN2 formed ectopically in the male brain, whereas pMN2 in the female brain remained unaffected (Fig 6). Thus, male-specific DsxM eliminates female-specific pMN2 in the male brain.

### A pC2l male homolog initiates male courtship

**Fig 7:**
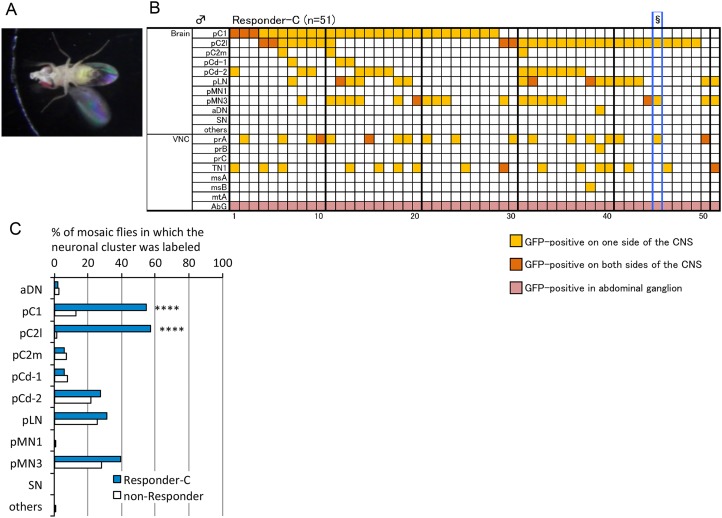
Activation of pC1 and pC2l neurons induces courtship in males. Male flies that carry *dTrpA1*-expressing MARCM clones were tested for the occurrence of courtship behavior in response to temperature increases, followed by immunohistochemical identification of neurons that were activated in the behavioral assays. We judged that a male responded to a temperature increase by courtship behavior (“Responder-C”) only when it vibrated its wings, while displaying any additional courtship actions, i.e., licking, tapping, and abdominal bending for copulation, and excluded those flies that exhibited wing displays alone without any additional element of courtship behavior, from the count of males positive for courtship responses. (**A**) Courtship behavior induced by a temperature increase to 35°C in a male carrying MARCM clones expressing dTrpA1. Note that the male is vibrating a wing while licking by extending his proboscis. (**B**) Labeling pattern of GFP expression in MARCM males. Each vertical column represents the scores of a single fly. Neuronal classes in the brain indicated in the left-hand column are described in Fig 3B and S1 Fig and S1 Table and those in the VNC are described in Ref. 19. § in B indicates that the images shown in Fig 7A, S3G and S3H Fig, and S10 Movie are of this fly. (**C**) The incidence of GFP-labeling of the indicated neural cluster (signifying dTrpA1 expression) was compared between the Responder-C (n = 51) and the rest of the tested flies (non-Responder, n = 114). GFP-labeling, and thus dTrpA1-mediated activation of pC1 and pC2l but none of the other clusters, was significantly correlated with the occurrence of courtship behavior in mosaic males. **** p < 0.0001, by Fisher’s exact probability test.

Whereas pMN2 is a female-specific neuron without a male homologue, pC2l has a male counterpart. To assess the function of pC21 neurons in males, mosaic males with MARCM clones that express dTrpA1 and mCD8::GFP, as driven by *dsx* ^*GAL4*^ *(G)*, were subjected to behavioral assays and histological analysis to identify the neurons involved in courtship behavior. A previous study revealed that mosaic females carrying a masculinized P1 clone courted females with unilateral wing vibration, but did not show any other advanced courtship actions [28]. Therefore, in the present study, we selected male mosaic flies that exhibited advanced courtship actions such as tapping, licking, and abdominal bending, which were classified as the Responder-C group (“C” stands for courtship; Fig 7A). Artificial activation of pC2l neurons was significantly correlated with the execution of courtship in these males (Fig 7B and 7C, S2 Fig, S10 Movie). In addition, excitation of pC1, a *dsx-GAL4*-positive cluster, which contains *fru*-expressing P1 neurons that initiate male courtship behavior [28, 29, 30], correlated significantly with courtship behavior (Fig 7C). The P1 cluster is composed of 20 *fru*-expressing neurons, which are entirely included in the *dsx*-positive pC1 cluster of 54 cells. Notably, a significant proportion of Responder-C flies who were positive for pC2l were also negative for pC1 (Fig 7B), which supports the notion that pC2l by itself likely plays an important role in the execution of sexual behavior in males. It remains to be examined whether bilateral inhibition of pC2l prevents males from displaying advanced courtship actions.

## Discussion

*Drosophila* females extend the ovipositor in two distinct contexts, mating behavior and oviposition. In the present study, we demonstrated, by means of clonal manipulation of brain neurons, that apparently similar ovipositor extensions in these two behaviors are initiated by distinct classes of *dsx-GAL4* neurons in the brain, pC2l for mating-type extrusion in response to male courtship and pMN2 for oviposition in mated females.

pC2l is the first brain neuron identified as specifically involved in the regulation of extrusion in female mating behavior. pC2l is a sexually dimorphic neural cluster with *dsx-GAL4* expression, and its counterpart in males also plays an important role in executing male courtship behavior. Extrusion has been recognized as an important component of rejection behavior in fertilized females that exhibit reduced receptivity to courting males for days after copulation. The importance of the brain in controlling female receptivity has been inferred by the analyses of gynandromorphs, which tended to be unreceptive to courting males when the gynandromorphs had male cuticles in their head regions [26]. The brain foci responsible for receptivity were narrowed down, by mapping with internal mosaic markers, to a region in the dorsal anterior brain, which needed to be bilaterally female in gynandromorphs to express receptivity to courting males [31]. The cellular identities of neurons for receptivity were only recently revealed by the MARCM or intersectional approach of activating or inactivating a small number of neurons or by the analysis of mutants with reduced receptivity. These studies uncovered five groups of brain neurons that play decisive roles in elevating female receptivity: the Spin-D cluster composed of subsets of olfactory projection neurons [15], the intrinsic neurons in the SOG called the Spin-A cluster [15], the female counterpart of the pC1cluster, which, in males, includes the P1 subgroup of neurons that initiates courtship in males [14], the pCd cluster composed of *dsx*-expressing neurons in the lateral protocerebrum [14] and some neurons with somata near the lateral horn [16]. Whereas these five groups of brain neurons determine the level of female receptivity, in the present study pC21 neurons appeared to be specifically involved in extrusion. A frequent pause in locomotion is a sign of elevated receptivity in virgin females that received insistent courtship by a male [32]. A recent study identified a subset of *Abd-B*-expressing VNC neurons that specifically regulate pausing in courted virgin females [12]. Thus different components of receptivity-associated behavioral changes are controlled by distinct neural clusters, which are distributed in both the brain and VNC. These observations imply that the brain-VNC interplay is pivotal for the appropriate expression of receptiveness by a female. Indeed, our results on the neural control of extrusion support this view. For example, in our study, mating-type extrusion was observable only when the brain neurons were activated clonally or *in toto* in the absence of concomitant activation of the VNC neurons, suggesting that an inhibitory effect of VNC activities on the brain system prevents the brain neurons from triggering the mating-type extrusion program presumably via an ascending pathway. A few ascending interneurons are known to affect female mating behavior: two SAG neurons with somata in the abdominal ganglia send axons along the brain midline to the dorsal protocerebrum while forming en passant synapses in the SOG [13]; 4 *dsx/ET* ^*FLP250*^ neurons with somata in the abdominal ganglia project to the SOG [10]. Artificial inactivation of SAG neurons converts virgin-female behavior into a mated-female-type behavior, whereas artificial activation converts mated-female behavior into a virgin-type behavior [13]. *dsx/ET* ^*FLP250*^ neurons have effects opposite to those of SAG neurons [10]. While it has been demonstrated that these ascending neurons alter the level of receptivity [33, 34], it remains an open question whether they have any direct effect on the extrusion-inducing mechanism.

In this study, we identified a *dsx-GAL4* expressing neural cluster, pMN2, as a strong activator of the oviposition program in the brain. pMN2 is a female-specific *dsx-GAL4*-positive descending neuron, and its counterpart in males is lost by cell death during development. It has been recognized that both the brain and VNC play important roles in oviposition because decapitated females are able to lay eggs [35], yet mated females with ablation in the pars intercerebralis of the brain lay only a few eggs in the manner of virgin females [25]. Furthermore, based on the observation that some of the virgin gynandromorphs with a mosaic border in the head lay as many eggs as fertilized females do, it has been suggested that the brain contains a region that inhibits oviposition in virgin females [26]. Indeed, the present results are, in principle, compatible with these classic works, except that our MARCM-based mapping did not point to neurons in the pars intercerebralis as key players in triggering oviposition. Perhaps our approach focusing on *dsx*-positive cells failed to detect some *dsx*-negative cells with effects on oviposition, and the pars intercerebralis neurons might be among them.

We found a highly significant correlation between pMN2 stimulation and the execution of the oviposition program when this *dsx-GAL4*-positive cluster was clonally activated by MARCM, whereas massive activation of *dsx-GAL4*-positive neurons in the brain (and not of those in the VNC) did not induce the oviposition posture and egg-laying, although the latter manipulation inevitably activated pMN2. Instead, the massive activation of *dsx-GAL4* neurons in the brain (and not of those in the VNC) resulted in mating-type extrusion. This apparently paradoxical result provokes the inference that a subset of *dsx-GAL4* neurons in the brain prevents pMN2 from functioning when both of them are artificially activated simultaneously. An intriguing possibility is that the *dsx-GAL4* neurons for the induction of a mating program in which pC2l is potentially included inhibit the *dsx-GAL4* neurons for triggering the oviposition program in which pMN2 is likely included. The motor program for oviposition posture and egg laying is located in the VNC, because artificial activation of *dsx-GAL4*-positive cells only in the VNC as achieved by the use of *Otd-GAL80* or by decapitation resulted in adoption of the oviposition posture and egg laying. Under normal conditions, pMN2 probably makes the decision to lay eggs and turns on the VNC motor program.

In view of the importance of female decisions in mating and oviposition and of the strong selective pressures expected to act on the system, we expect that the neural circuitries underlying these behaviors are rather complicated. The present identification of two brain neurons that play central roles in female reproductive behaviors provides a promising entry point for the solid analysis of decision-making circuitries at the single cell resolution.

## Supporting Information

S1 Fig Projection patterns of *dsx*-expressing neurons. MARCM clones with specific labeling by the *dsx* ^*GAL4*^ *(G)*-driven reporter, mCD8-GFP. Somata of *dsx* ^*GAL4*^ *(G)*-labeled neurons are indicated by yellow arrows. Male-specific and female-specific projections are indicated by green and magenta arrowheads, respectively. The SN neuron is circled to distinguish it from other co-labeled neurons. Brains and VNCs were doubly stained with anti-GFP (or anti-mCD8) (green) and nc82 mAb (magenta).
(TIFF)

S2 Fig Labeling pattern of GFP expression in flies subjected to behavioral MARCM. The data sheets showing the composition of labeled neurons in every mosaic female (**A, B, C**) and male (**D, E**). The genotypes of the flies are *y hs-flp/+(Y);G13 UAS-mCD8*::*GFP/G13 Tub-GAL80;dsx* ^*GAL4*^ *(G)/UAS-dTrpA1*.
(TIFF)

S3 Fig GFP-labeling in the brains and VNCs of flies subjected to behavioral MARCM. (**A, B**) The brains and VNC of a female indicated by an asterisk (*) in Fig 4B. The female laid an egg upon a temperature increase, as shown in Fig 4A and S8 Movie. pMN2 (arrow in **A**, **B**) and several ventral neurons (**B**) are labeled. (**C, D**) The brain and VNC of a female indicated by a pound sign (#) in Fig 4D. The female extruded the ovipositor upon a temperature increase, as shown in Fig 4C and S9 Movie. pC2l, pCd-2 (arrows in **C**), and several ventral neurons (**D**) are labeled. (**E, F**) The brain and VNC of a female that extruded the ovipositor upon a temperature increase. pC2l clones are labeled bilaterally (arrows in **E**). Several ventral neurons are also labeled (**F**). (**G, H**) The brain and VNC of a male indicated by § in Fig 7B. This male exhibited courtship behavior upon a temperature increase, as shown in Fig 7A and S10 Movie. pC2l and pMN3 (arrows in **G**) are labeled in addition to thoracic prA neurons (an arrow in **H**) and several ventral neurons (**H**). The genotype of the flies is *y hs-flp/+(Y);G13 UAS-mCD8*::*GFP/G13 Tub-GAL80;dsx* ^*GAL4*^ *(G)/UAS-dTrpA1*. Brains and VNCs were doubly stained with anti-GFP (green) and nc82 mAb (magenta).
(TIFF)

S1 Table Classification of *dsx*-expressing neurons based on *dsx* ^*GAL4*^ labeling in the adult brain. The number of cells composing a cluster is counted in a hemi-brain. Six individuals (12 hemibrains) were counted in both sexes (F: females; M: males).
(DOCX)

S2 Table The number of MARCM mosaic flies analyzed in this report. * The flies in which the CNS was lost or damaged during manipulations and those without labeled CNS neurons were not included.
(DOCX)

S3 Table The number of Responder-O and Responder-M flies in four fly groups classified by the presence and absence of dTrpA1 expression in pMN2 and pC2l neurons (DOCX)

S1 Movie Ovipositor extrusion displayed by a mated wild-type female. A mated wild-type female (CS strain) shows rejection behavior with prolonged extrusion of the ovipositor towards the courting wild-type male (CS strain).
(MOV)

S2 Movie A wild-type female engaging in oviposition. A wild-type female (CS strain) extends her ovipositor and lays eggs on a grape-juice medium.
(MOV)

S3 Movie Oviposition posture induced by a temperature increase in a dsx^*GAL4*^ *>dTrpA1* female. Oviposition posture is induced in a female with the genotype *G13 UAS-mCD8*::*GFP; dsx* ^*GAL4*^ *(G) UAS-dTrpA1 /TM6b Hu Tb* by an increase in temperature from 22°C to 29°C.
(MOV)

S4 Movie Oviposition induced by a temperature increase in a dsx^*GAL4*^ *>dTrpA1* female. Oviposition is induced in a female with the genotype *G13 UAS-mCD8*::*GFP; dsx* ^*GAL4*^ *(G) UAS-dTrpA1 /TM6b Hu Tb* by an increase in temperature from 22°C to 29°C.
(MOV)

S5 Movie Oviposition posture and mating-type extrusion of the ovipositor observed in a dsx^*GAL4*^ *>dTrpA1* female in the presence of a courting male. A mated female with the genotype *G13 UAS-mCD8*::*GFP; dsx* ^*GAL4*^ *(G) UAS-dTrpA1 /TM6b Hu Tb* exhibits the oviposition posture with ovipositor extension by an increase in temperature at 29°C. In addition, the mating-type extrusion toward the male is induced in response to male courtship.
(MOV)

S6 Movie Mating-type extrusion of the ovipositor induced by a temperature increase in a female expressing dTrpA1 in *dsx*-positive neurons in the brain. Mating-type extrusion of the ovipositor is induced in a mated female with the genotype of *UAS>stop>dTrpA1-myc/Otd-nsl*:*FLP; dsx* ^*GAL4*^ *(G) / +* by an increase in temperature to 32°C.
(MOV)

S7 Movie Oviposition posture induced by a temperature increase in a female expressing dTrpA1 in *dsx*-positive neurons outside the brain. The oviposition posture is induced in a mated female with the genotype of *Tub>stop>GAL80/Otd-nsl*:*FLP; dTrpA1 dsx* ^*GAL4*^ *(G) / +* by an increase in temperature to 32°C.
(MOV)

S8 Movie Oviposition induced by a temperature increase in a female carrying *dsx* ^*GAL4*^ *>dTrpA1* MARCM clones. The oviposition posture/egg-laying is induced in a female (the genotype is *y hs-flp/+; G13 UAS-mCD8*::*GFP/G13 Tub-GAL80; dsx* ^*GAL4*^ *(G)/UAS-dTrpA1)* that carries dTrpA1-expressing MARCM-clone neurons at 35°C.
(MOV)

S9 Movie Mating-type extrusion of the ovipositor induced by a temperature increase in a female carrying *dsx* ^*GAL4*^ *>dTrpA1* MARCM clones. Mating-type extrusion of the ovipositor is induced in a female (the genotype is *y hs-flp/+; G13 UAS-mCD8*::*GFP/G13 Tub-GAL80; dsx* ^*GAL4*^ *(G)/UAS-dTrpA1)* bearing dTrpA1-expressing MARCM-clone neurons at 35°C.
(MOV)

S10 Movie Courtship behavior induced by a temperature increase in a male carrying *dsx* ^*GAL4*^ *>dTrpA1* MARCM clones. Courtship behavior is induced by a temperature increase to 35°C in a male with the genotype of *y hs-flp/Y; G13 UAS-mCD8*::*GFP/G13 Tub-GAL80; dsx* ^*GAL4*^ *(G)/UAS-dTrpA1*. The fly carrying dTrpA1-expressing MARCM-clone neurons exhibits a wing display and proboscis extension (licking) at 35°C.
(MOV)
