# Supplementary figures and images for: Drosophila Ovipositor Extension in Mating Behavior and Egg Deposition Involves Distinct Sets of Brain Interneurons
Source: PLoS One. 2015 May 8;10(5):e0126445. doi: 10.1371/journal.pone.0126445 (PMC4425497; doi:10.1371/journal.pone.0126445)

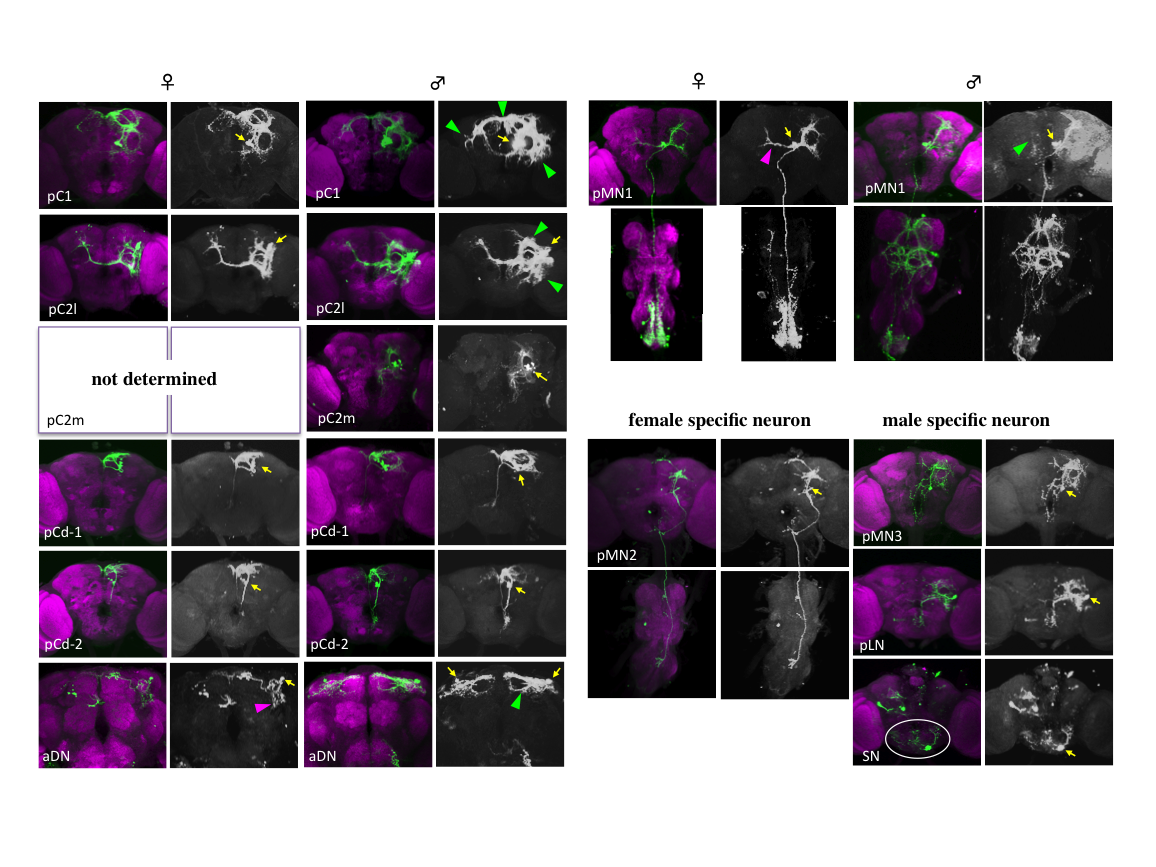

Supplement: S1 Fig — MARCM clones with specific labeling by the dsx GAL4 (G)-driven reporter, mCD8-GFP. Somata of dsx GAL4 (G)-labeled neurons are indicated by yellow arrows. Male-specific and female-specific projections are indicated by green and magenta arrowheads, respectively. The SN neuron is circled to distinguish it from other co-labeled neurons. Brains and VNCs were doubly stained with anti-GFP (or anti-mCD8) (green) and nc82 mAb (magenta). (TIFF) [file pone.0126445.s001.tiff]

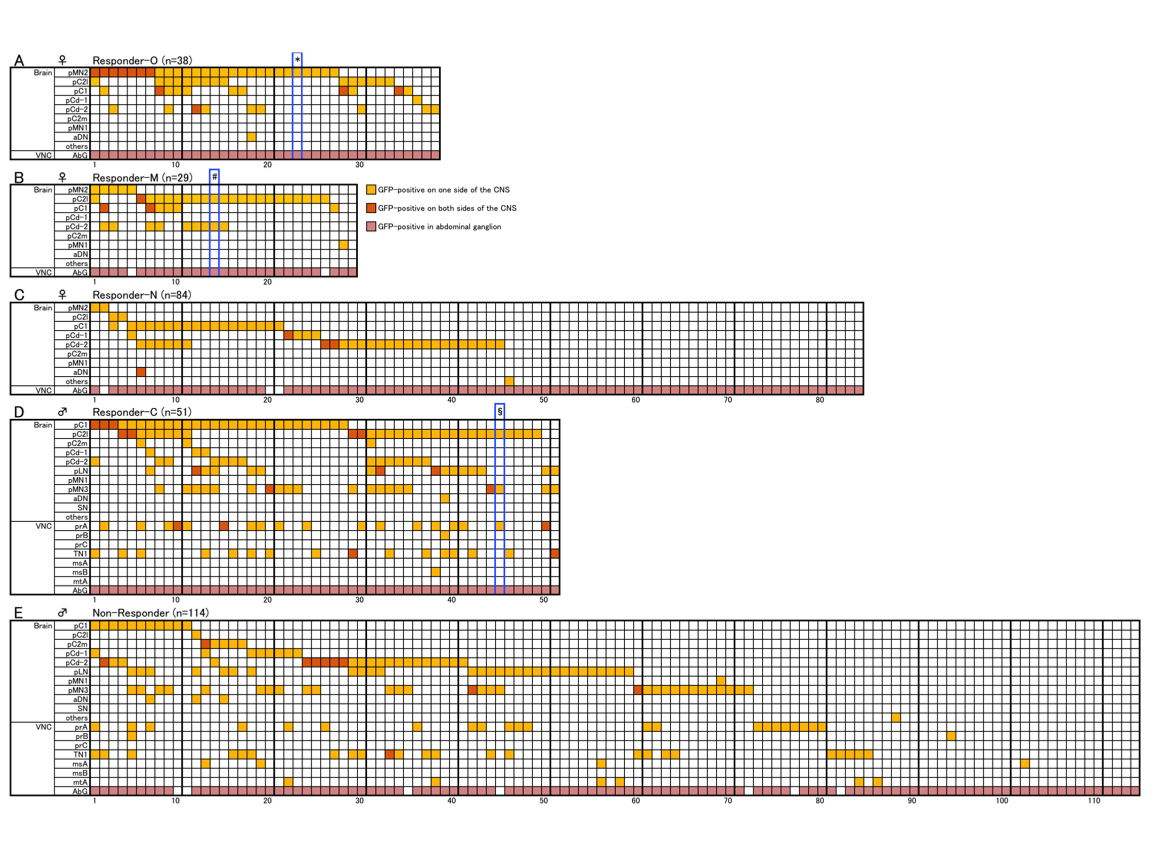

Supplement: S2 Fig — The data sheets showing the composition of labeled neurons in every mosaic female (A, B, C) and male (D, E). The genotypes of the flies are y hs-flp/+(Y);G13 UAS-mCD8::GFP/G13 Tub-GAL80;dsx GAL4 (G)/UAS-dTrpA1. (TIFF) [file pone.0126445.s002.tiff]

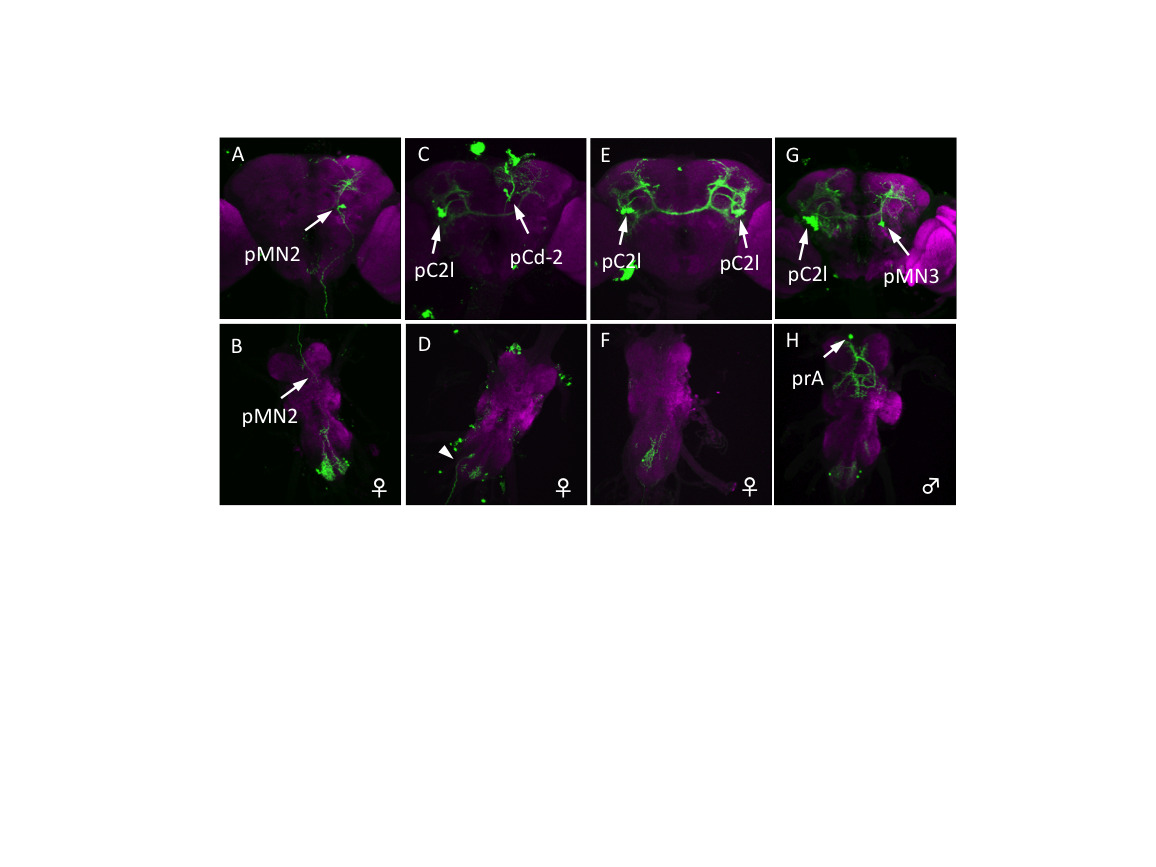

Supplement: S3 Fig — (A, B) The brains and VNC of a female indicated by an asterisk (*) in Fig 4B. The female laid an egg upon a temperature increase, as shown in Fig 4A and S8 Movie. pMN2 (arrow in A, B) and several ventral neurons (B) are labeled. (C, D) The brain and VNC of a female indicated by a pound sign (#) in Fig 4D. The female extruded the ovipositor upon a temperature increase, as shown in Fig 4C and S9 Movie. pC2l, pCd-2 (arrows in C), and several ventral neurons (D) are labeled. (E, F) The brain and VNC of a female that extruded the ovipositor upon a temperature increase. pC2l clones are labeled bilaterally (arrows in E). Several ventral neurons are also labeled (F). (G, H) The brain and VNC of a male indicated by § in Fig 7B. This male exhibited courtship behavior upon a temperature increase, as shown in Fig 7A and S10 Movie. pC2l and pMN3 (arrows in G) are labeled in addition to thoracic prA neurons (an arrow in H) and several ventral neurons (H). The genotype of the flies is y hs-flp/+(Y);G13 UAS-mCD8::GFP/G13 Tub-GAL80;dsx GAL4 (G)/UAS-dTrpA1. Brains and VNCs were doubly stained with anti-GFP (green) and nc82 mAb (magenta). (TIFF) [file pone.0126445.s003.tiff]
