# Supplementary material for: Drosophila Ovipositor Extension in Mating Behavior and Egg Deposition Involves Distinct Sets of Brain Interneurons
Source: PLoS One. 2015 May 8;10(5):e0126445. doi: 10.1371/journal.pone.0126445 (PMC4425497; doi:10.1371/journal.pone.0126445)
Supplement: S1 Table — The number of cells composing a cluster is counted in a hemi-brain. Six individuals (12 hemibrains) were counted in both sexes (F: females; M: males). (DOCX) [file pone.0126445.s004.docx]

**S1 Table. Classification of *dsx*-expressing neurons based on *dsx^GAL4^* labeling in the adult brain**

| Lee et al.（2002） | Rideout et al. (2010) | Robinett et al. (2010) | This report |  |  |  |  |  |  |  |
| --- | --- | --- | --- | --- | --- | --- | --- | --- | --- | --- |
|  |  |  |  | F |  |  |  | M |  |  |
|  |  |  |  | mean | ± | s.e. |  | mean | ± | s.e. |
| dsx-pC1 | dsx-pC1 | pC1 | pC1 | 26.8 | ± | 1.3 |  | 53.8 | ± | 1.2 |
| dsx-pC2 | dsx-pC2 | pC2l | pC2l | 21.8 | ± | 0.8 |  | 35.9 | ± | 1.7 |
|  |  | pC2m | pC2m | 3.9 | ± | 0.3 |  | 30.8 | ± | 1.3 |
|  |  | pLN | pLN | 0 |  |  |  | 1 |  |  |
|  | dsx-pC3 | pCd | pCd-1 | 8.4 | ± | 0.4 |  | 11.8 | ± | 0.7 |
|  |  |  | pCd-2 | 3.8 | ± | 0.2 |  | 4.1 | ± | 0.3 |
|  |  | pMN1 | pMN1 | 1 |  |  |  | 1 |  |  |
|  |  | pMN2 | pMN2 | 1 |  |  |  | 0 |  |  |
|  |  |  | pMN3 | 0 |  |  |  | 1 |  |  |
| dsx-aDN | dsx-aDN | aDN | aDN | 2.0 | ± | 0.2 |  | 2.1 | ± | 0.1 |
| dsx-SN | dsx-SN | SN | SN | 0.2 | ± | 0.1 |  | 1 |  |  |
| total |  |  |  | 68.9 |  |  |  | 142.5 |  |  |

The number of cells composing a cluster is counted in a hemi-brain.

Six individuals (12 hemibrains) were counted in both sexes (F: females; M: males).
