# Supplementary material for: Drosophila Ovipositor Extension in Mating Behavior and Egg Deposition Involves Distinct Sets of Brain Interneurons
Source: PLoS One. 2015 May 8;10(5):e0126445. doi: 10.1371/journal.pone.0126445 (PMC4425497; doi:10.1371/journal.pone.0126445)
Supplement: S2 Table — * The flies in which the CNS was lost or damaged during manipulations and those without labeled CNS neurons were not included. (DOCX) [file pone.0126445.s005.docx]

**S2 Table. The number of MARCM mosaic flies analyzed in this report**

No. of mosaic flies examined*

female Responder 67

Responder-O (oviposition-type extension) 38

Responder-M (mating-type extrusion) 29

Responder-N 84

　　　　　　　　 　　 total 151

male Responder-C 　 51

non-Responder 114

total 165

* The flies in which the CNS was lost or damaged during manipulations and those without labeled CNS neurons were not included.
