# Supplementary material for: Drosophila Ovipositor Extension in Mating Behavior and Egg Deposition Involves Distinct Sets of Brain Interneurons
Source: PLoS One. 2015 May 8;10(5):e0126445. doi: 10.1371/journal.pone.0126445 (PMC4425497; doi:10.1371/journal.pone.0126445)
Supplement: S3 Table — (DOCX) [file pone.0126445.s006.docx]

**S3 Table. The number of Responder-O and Responder-M flies in four fly groups classified by the presence and absence of dTrpA1 expression in pMN2 and pC2l neurons**

pMN2 pC2l Responder-O Responder-M total

+ - 18 4 22

- + 6 21 27

+ + 9 1 10

- - 5 3 8

total 38 29 　 67
